# The shift in ocular dominance from short-term monocular deprivation exhibits no dependence on duration of deprivation

**DOI:** 10.1038/s41598-018-35084-1

**Published:** 2018-11-20

**Authors:** Seung Hyun Min, Alex S. Baldwin, Alexandre Reynaud, Robert F. Hess

**Affiliations:** McGill Vision Research, Dept, Ophthalmology, McGill University, Montreal, Quebec, Canada

## Abstract

Deprivation of visual information from one eye for a 120-minute period in normal adults results in a temporary strengthening of the patched eye’s contribution to binocular vision. This plasticity for ocular dominance in adults has been demonstrated by binocular rivalry as well as binocular fusion tasks. Here, we investigate how its dynamics depend on the duration of the monocular deprivation. Using a binocular combination task, we measure the magnitude and recovery of ocular dominance change after durations of monocular deprivation ranging from 15 to 300 minutes. Surprisingly, our results show that the dynamics are of an all-or-none form. There was virtually no significant dependence on the duration of the initial deprivation.

## Introduction

The inputs from the two eyes are segregated into ocular dominance columns in the input layers of cortical area V1. They are only later combined in the more superficial layers^1^. Any imbalance in the inputs from the two eyes can lead to competitive changes. These then affect the relative width of the ocular dominance column^1^. Neuroplastic changes after long-term monocular occlusion have been used as an index of ocular dominance plasticity in animals^2^ and man^3–5^. These findings have helped define “the critical period” for visual development^6^. It is well-established that monocular deprivation during the critical period can permanently reduce the functioning of the deprived eye and shift ocular dominance in favour of the unpatched eye^6^. With this principle in mind, physicians for the past 250 years have recommended monocular occlusion of the fixing eye as treatment for children with amblyopia. Beyond the critical period, monocular occlusion of the fixing eye becomes ineffective for amblyopia treatment. However recent studies have shown that monocular deprivation for as little as 120 minutes in the adult *strengthens* the deprived eye’s contribution to the binocular percept^4,5,7^, an opposite finding to previous studies. This finding is surprising for two reasons. First, it shows that there is residual neural plasticity in the adult’s primary visual cortex. Second, the effect of short-term monocular deprivation in adults strengthens the opposite eye to that of long-term monocular deprivation in early life. In adults, the contribution of the patched eye increases after a brief period (a few hours) of deprivation^4^, whereas in young animals the patched eye loses function after long-term monocular deprivation (days)^2^. The results from studies of short-term monocular deprivation-induced ocular dominance changes show that the effect is transient in nature. The majority of the recovery occurs over a period of 30–90 minutes in adults^4,5^. The return to baseline of this neuroplastic change suggests that there are homeostatic mechanisms^8^ maintaining the balance of ocular dominance. Occluding one eye can only temporarily introduce an imbalance before the original balance is restored.

There are many approaches that can be used to quantify the contribution of each eye to binocular vision. Several of these have been used to measure the shift in ocular dominance in adults as the result of a short-term disruption to the input of one eye. The typical protocol is to measure the baseline balance between the two eyes (e.g. a ratio of each eye’s influence) and then measure the balance following a period of monocular patching. The patching effect is quantified by taking the change in the balance between the two measurements. The tasks previously used include binocular combination tasks^4^ and binocular competition (e.g. rivalry) tasks^3,5^. In terms of binocular combination, different stimuli have been used including interocular phase^4,9^, interocular perceived contrast^4^, dichoptic global motion coherence^4^ and an edge-detection task measuring both fusion and suppression^10^.

In binocular rivalry, incompatible stimuli are presented to each eye which cause the inputs from each eye to compete rather than combine (fuse). Subjects are asked to report on the relative durations of each perceived stimulus. A change in eye dominance is indicated by a shift in the relative duration of each eye’s percept. In binocular combination tasks, two fusible stimuli are shown to each eye. The influence of the two eyes in binocular vision is measured by obtaining information from the subject about the fused percept (where the input from each eye would bias the subject towards two different percepts). Although these two approaches (binocular rivalry and binocular combination) both support the view that short term monocular deprivation in the adult shifts ocular dominance in favour of the previously patched eye, the neural mechanisms involved in each task may be different. This is because binocular rivalry involves stimuli that are likely represented by separate neural populations (i.e. neurons with different preferred orientations), whereas combination tasks involve stimuli that are likely to activate a common neural population (i.e. neurons with the same orientation preferences). For example, co-oriented gratings seen by the two eyes would be expected to stimulate an overlapping population of simple cells in primary visual cortex. Recently, uncorrelated individual differences have been shown for short-term monocular deprivation for cross-oriented and co-oriented dichoptic masking^11^. This is consistent with there being a different neural substrate for ocular dominance changes after monocular deprivation, as revealed by binocular rivalry and combination tasks.

Besides psychophysical techniques^4,5,9,12^ electrophysiological and neuroimaging techniques have also demonstrated the robust effect of monocular deprivation^13–15^. However, the majority of studies, be they psychophysical or electrophysiological, have only examined durations of monocular deprivation of between 120–150 minutes. So, although we have a good idea of the recovery of the effect (at least for this time scale of deprivation), we have no idea of how this varies with the duration of deprivation. In this study, we have examined the duration dependence of the effects of monocular deprivation-induced changes in ocular dominance. To do this we have measured changes in ocular dominance in adults across different time scales of monocular deprivation; a short-time scale from 15 to 30 minutes (first experiment) and a longer-time scale from 60 to 300 minutes (second experiment). In each of the experiments we measured the eye dominance using the phase combination task^4^ (see methods), patched one eye for variable periods of time (15–300 min) and re-measured the eye balance at each of a number of time points (0, 3, 6, 12, 24, 48, 60 and 96 min) after monocular deprivation. We find that there is at best only a very weak relationship between patching duration and ocular dominance plasticity. A 20-fold increase in the deprivation duration results in the strength of the dominance change only increasing by a 25%. Also, the recovery of the patching effect seems to be quite similar across all deprivation durations, implying that there may not be duration dependence in the recovery. This finding implies that this homeostatic process has unusual dynamics being, to a first approximation, an all-or-none phenomenon.

## Result

### Short duration of monocular deprivation (15–30 minutes)

**Figure 1:**
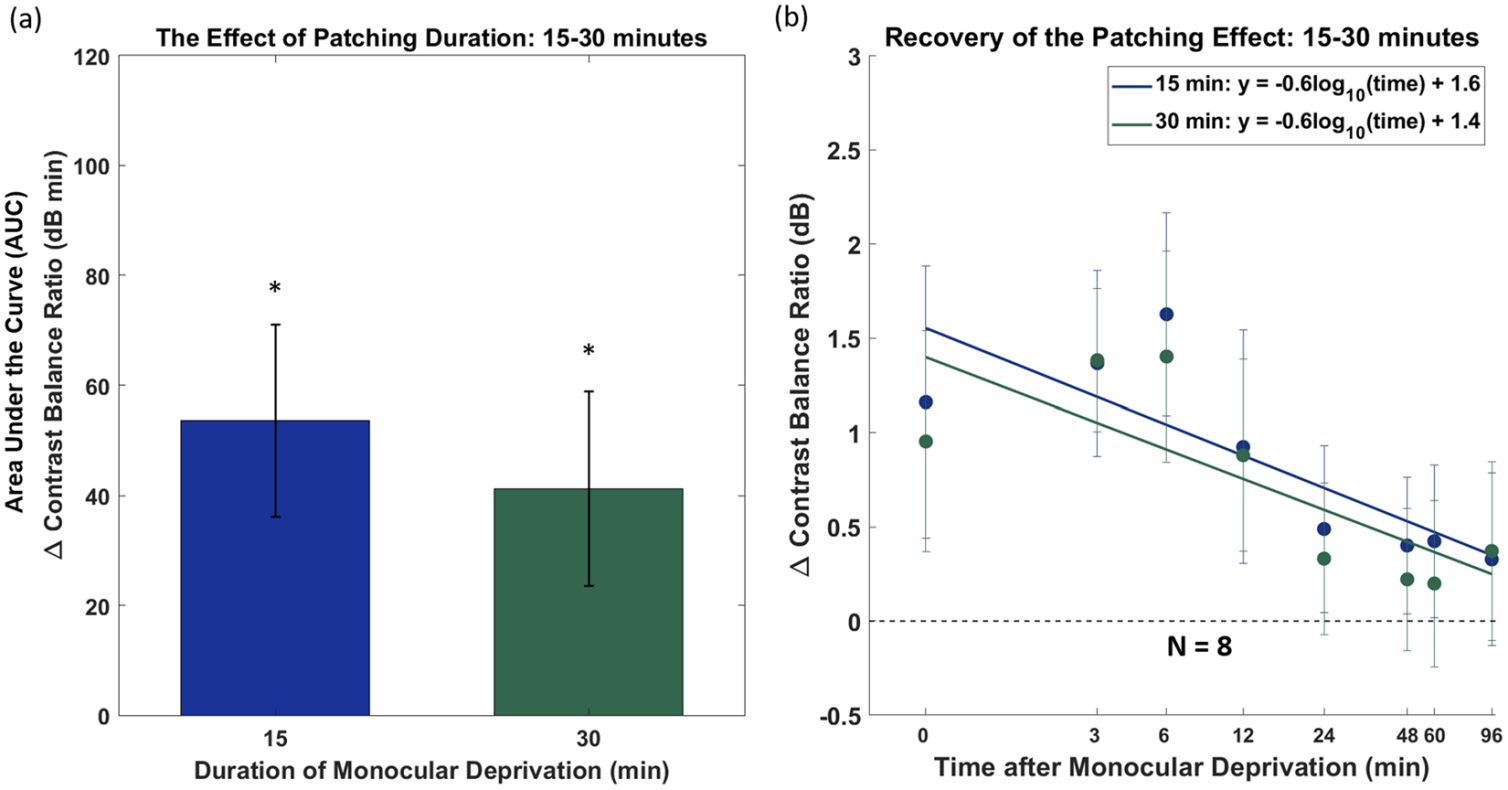
Monocular deprivation for 15 and 30 minutes on eight subjects. (**a**) Area under the curve calculated from (**b**). The error bars represent standard errors across the AUC ∆ contrast balance ratio of subjects. Measurements that are significantly different (Wilcoxon Signed Rank test) from baseline are indicated; *p < 0.05. (**b**) Recovery of the patching effect over time after patch removal on log/log scaled axes. Each point represents the change in eye dominance as a function of the time after monocular deprivation. The x-axis values represent the time-points of post-patching measurement. The error bars represent standard errors.

In the first experiment, we measured eye dominance using the binocular phase combination task^4^ for two durations of monocular occlusion: 15 and 30 minutes. We plotted the data on semi-log coordinates and fitted them with a straight line (i.e. an exponential function) as this is consistent with the form of the recovery in previous studies using the phase combination procedure^9,16^. The changes in eye dominance relative to baseline for the cohort of eight subjects are presented in Fig. 1b. If the patched eye became stronger, ∆ contrast balance ratio would be positive. Since the effect of patching was plotted relative to baseline, ∆ contrast balance ratio of 0 would represent the pre-patch baseline.

The averaged eye dominance change induced by patching durations of 15 and 30 minutes recovered back to baseline in 24 minutes (shown by the recovery slopes in Fig. 1b). The Wilcoxon Signed Rank Test showed no significant difference between the recovery slopes of 15 and 30 minutes (p = 0.95). The patching effect peaked at between 0 and 7 minutes after patch removal. The peak imbalance induced by the patching was 1.5 dB. When shown stimuli of the same contrast to their two eyes, the subjects responded as if there was a 19% difference in contrast.

Figure 1a shows the area under the curve (AUC) of Fig. 1b (summation of Δ contrast balance ratio from 0 to 96 minutes after monocular deprivation). To capture both the magnitude and duration of the effect we computed the AUC Δ contrast balance ratio. The higher the AUC, the stronger the patching effect for the patched eye. The Wilcoxon Signed Rank Test showed no significant difference between the calculated AUCs for the two patching durations (p = 0.95).

### Longer duration of monocular deprivation (60–300 minutes)

**Figure 2:**
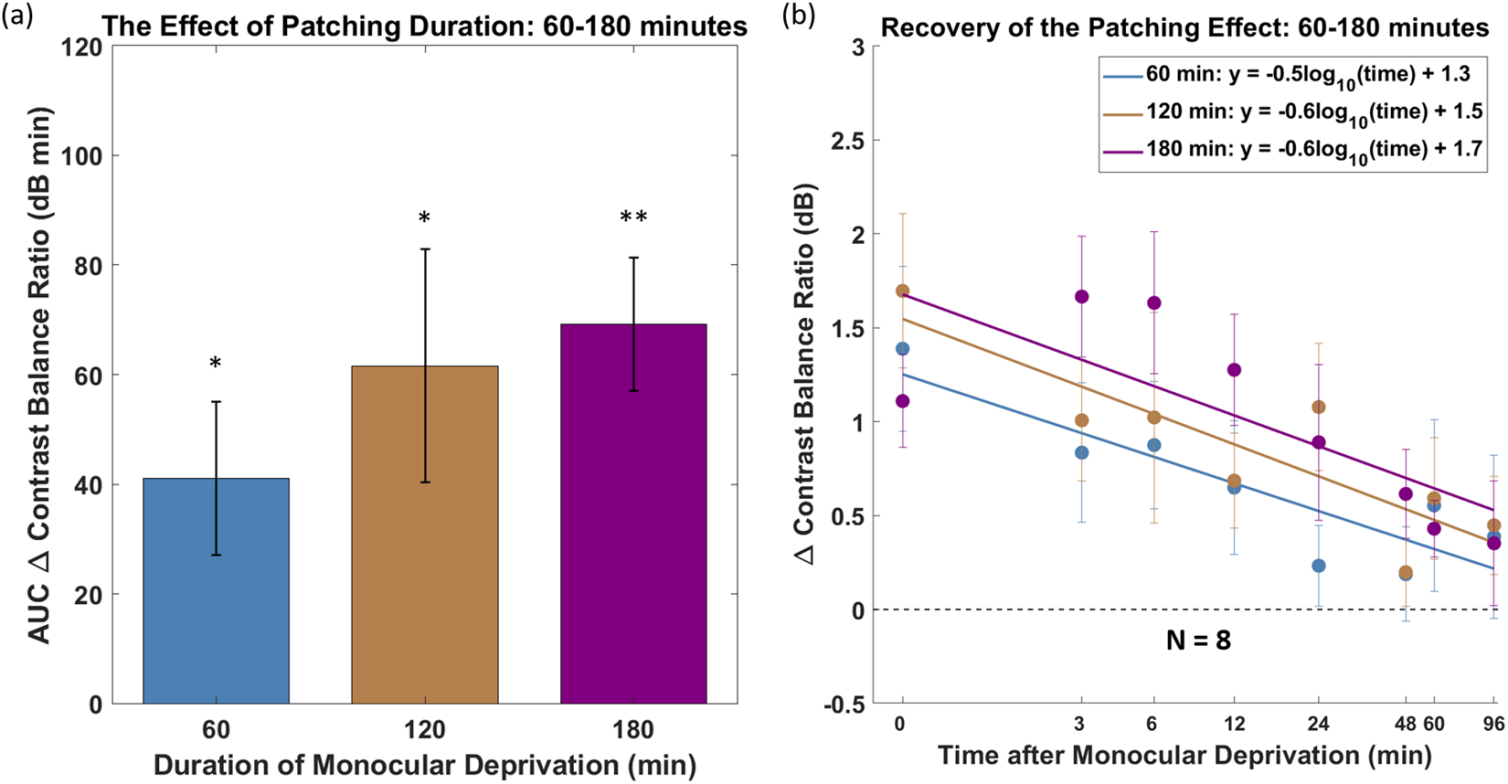
Monocular deprivation for 60, 120 and 180 minutes on another cohort of eight subjects. (**a**) Area under the curve calculated from (**b**). The error bars represent standard errors across the AUC ∆ contrast balance ratio of subjects. Measurements that are significantly different (Wilcoxon Signed Rank test) from baseline are indicated; **p < 0.01, *p < 0.05. (**b**) Recovery of the patching effect over time after patch removal on log/log scaled axes. Each point represents the change in eye dominance as a function of time after monocular deprivation. The x-axis values represent the time-points of post-patching measurement. The error bars represent standard errors.

In the second experiment, we measured ocular dominance changes on another cohort of eight subjects for a range of longer durations, namely 60, 120 and 180 minutes. In Fig. 2b, recovery curves are shown on log/log scaled axes and fitted with straight lines. The slopes of these fits are about −0.5 and −0.6 on log/log scaled axes (Fig. 2b). The Wilcoxon Signed Rank Test showed that the slopes are not statistically different between 60 and 120 minutes (p = 0.84), 60 and 180 minutes (p = 0.95) and 120 and 180 minutes (p = 0.95). Although there is a trend that longer durations of patching are associated with greater areal effects (AUC Δ contrast balance ratio), this was not statistically significant. The Wilcoxon Signed Rank Test showed no significant difference between the AUCs of 60 and 120 minutes (p = 0.38), as well as between 120 and 180 minutes (p = 0.74). There was also no significant difference between the AUCs of 60 and 180 minutes (p = 0.25).

**Figure 3:**
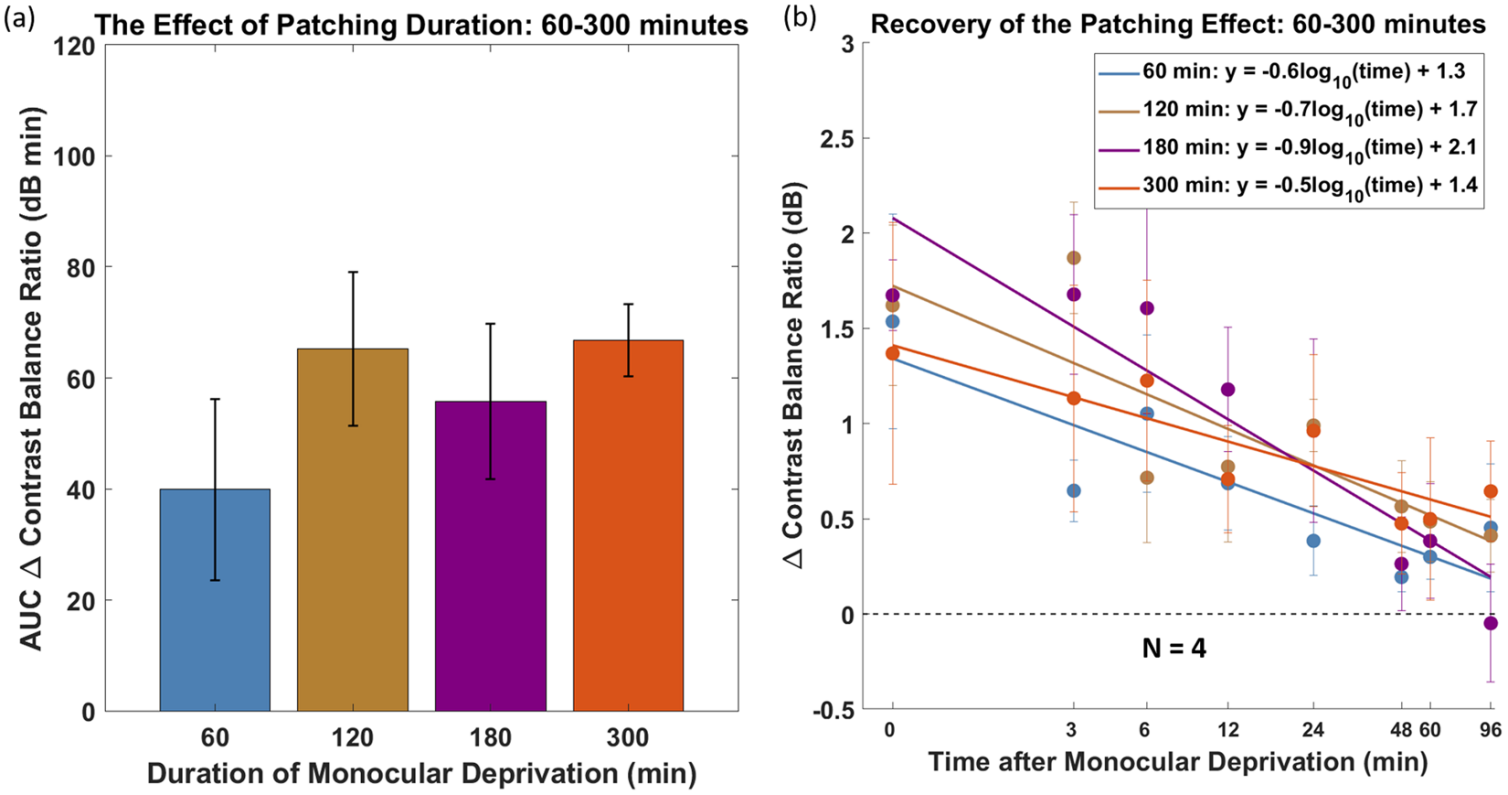
Monocular deprivation for 60, 120, 180 and 300 minutes on *four* of the eight subjects in the second cohort. (**a**) Area under the curve calculated from (**b**). The error bars represent standard errors across the AUC ∆ contrast balance ratio of subjects. (**b**) Recovery of the patching effect over time after patch removal on log/log scaled axes. Each point represents the change in eye dominance as a function of the time after monocular deprivation. The x-axis values represent the time-points of post-patching measurement. The error bars represent standard errors.

In the third experiment, four of the cohort above were also patched for 300 minutes (Fig. 3). Although there is a trend that the patching effect from 60 minutes patching is less (i.e. Figure 3a-AUC ∆ contrast balance ratio), the Wilcoxon Signed Rank test showed no significant difference between the AUCs in 60 and 300 minutes of patching (p = 0.13). The AUCs of the patching effect at 60 and 300 minutes duration were also similar.

**Figure 4:**
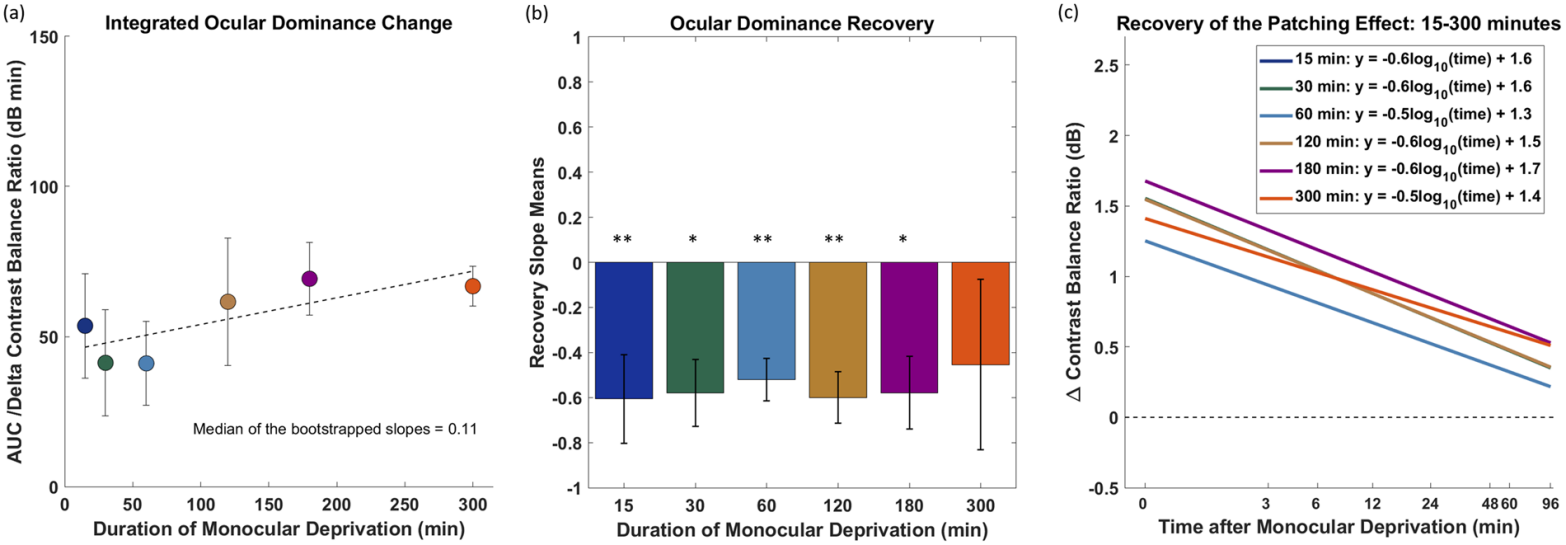
Summary of integrated ocular dominance changes from monocular deprivation of varying durations (**a**), their associated recovery times (**b**) and recovery of the patching effect (**c**). The first cohort of eight subjects performed 15 and 30 minutes of patching. The second cohort of eight subjects performed 60, 120, and 180 minutes of patching. Four of the second cohort performed 300 minutes of patching. (**a**) AUCs ∆ contrast balance ratio of all subjects is summarized in this figure. The error bars represent standard errors across the AUC ∆ contrast balance ratio of subjects. (**b**) Recovery slope means that are significantly different (Wilcoxon Signed Rank test) from 0 are indicated; **p < 0.01, *p < 0.05. The error bars represent standard errors of the recovery slopes across subjects. (**c**) Recovery of the patching effect on log/log scaled axes in all durations. The plots represent best fit lines to all data in each time point after monocular deprivation. The plots of 15 and 120 minutes are superimposed so only the plot of 120 minutes is visible.

The general summary of all results is shown in Fig. 4a. Although we found no significant effect of patching duration on the magnitude of the ocular dominance change, we are able to rule out the possibility of there being a relationship at all. When taking into account the data from all the patching durations examined in this study, there does seem to be a trend for longer durations to have slightly larger areal effects. To quantify this global trend, we derived bootstraps of the slope parameter (median = 0.1127) of the best fitting linear function (Fig. 4a- solid line) and found no overlap between the slope value of 0 and the 95% confidence interval (0.11 [95% CI −0.08, +0.08]) of the fitted slope. This suggests that there is a small but significant global trend when all patching durations are taken together. However, it is worth noting that a one-way analysis of variance (ANOVA) showed no significant difference (p = 0.75) between the areal effects of all patching durations.

It could be that for the sample size used here, the data we collected failed to represent a (small) real underlying effect (type II error). From looking at the statistical power of the Wilcoxon Signed Rank test^15^ and the standard deviation of the data we collected, we estimated the magnitude of the underlying effect size that would have to exist for us to be able to reliably detect it. For simplicity we only performed this analysis for the AUC comparisons. We determined that the difference between the effects for two patching durations would have to be around 45 dB minutes. This is approximately the magnitude of the patching effect that we find for the shorter patching durations compared to baseline. This means that reliably finding a significant difference between 15 and 180 minutes would, for example, require that the patching effect for 180 minutes to be at least 90 dB minutes. Another way of thinking about this, is to first assume that the small areal effect we find between 15 and 180 minutes is real. In this case we would need an excess of 300 participants for such an effect to reach statistical significance. Therefore, while we cannot conclude that there is no effect of patch duration on ocular dominance plasticity, we can conclude that any such effect is very small.

We were interested in whether there was a relationship between the patching duration and the rate of recovery in the patching effect. After calculating the recovery slope (linear fits in log/log scaled space) means of all subjects for each condition (Fig. 4b) we performed the Wilcoxon Signed Rank test across conditions. It showed no significant difference between the recovery slopes of 60 and 120 minutes of patching (p = 0.55), neither between 60 and 180 minutes of patching (p = 0.84) or between 120 and 180 minutes of patching (p > 0.05). We also performed a one-way ANOVA across all durations and found no statistically significant difference (p = 0.99). The lack of a significant difference across different patching durations implies that longer durations of monocular deprivation do not result in a slower recovery of ocular dominance to baseline.

## Discussion

We used a binocular phase combination task to measure changes in eye dominance from different durations of monocular deprivation. We employed six different durations spanning 15 to 300 minutes. We tracked changes in visual recovery over 96 minutes following monocular deprivation. We had expected that there would be a strong relationship between both the magnitude and recovery duration of visual plasticity and the duration of monocular deprivation. However, we observed only a minimal increase in the magnitude of the ocular dominance change resulting from 15 to 300 minutes of monocular deprivation; a 20-fold increase in the duration of deprivation resulted in only a 25% difference in the ocular dominance effect. Also, we found that regardless of the patching duration, the neuroplastic recovery after monocular occlusion returned to baseline within 96 minutes. This implies that these changes reflect a homeostatic mechanism^8^ that responds instantaneously in an all-or-none fashion to a disrupted monocular input of variable duration.

The other end-point measure used to measure changes in ocular dominance occurring as the result of monocular deprivation has been binocular rivalry. Lunghi *et al*. used that task when they first demonstrated that monocular occlusion changed the sensory eye balance in favour of the patched eye. In their study, monocular deprivation of 150 minutes was employed^5^. Several groups since then have corroborated the short-term monocular deprivation effect in adults using various psychophysical and neuroimaging techniques^4,5,9,11–14^. Although the bulk of literature has only examined durations of 120 to 150 minutes, some studies have provided information on shorter periods of monocular deprivation using continuous flash suppression. Kim *et al*., examined durations as short as 15 minutes and showed that monocular deprivation from continuous flash suppression could induce an effect comparable to that observed after the same duration of monocular deprivation using a diffuser^17^. They showed a significant change in dominance after only 3 minutes of monocular deprivation. However, no study has systematically investigated the relationship between the duration of monocular deprivation and either the magnitude or duration of subsequent ocular dominance changes.

This finding of a virtually all-or-none response of the binocular visual system to an imbalance in the input from the two eyes of variable duration is unexpected. Early in life there is a critical period of development during which the visual system is most plastic. During the critical period the duration of monocular deprivation affects both the magnitude and the recovery time of changes in ocular dominance^18^. Therefore, adult neuroplastic changes in ocular dominance measured here are not simply a reduced version of their counterparts in early life: not only is the effect in the opposite direction (i.e. strengthening of the patched eye in adults, a weakening of the patched eye in children) but the dynamics are also fundamentally different. Furthermore, the difference here may not simply be between juvenile and adult forms of plasticity because “adaptation” in the adult which is also known to alter sensitivity in the short term through homeostatic mechanisms^8^ also exhibits different properties to that of adult ocular dominance plasticity. Adaptation after-effects have been shown for a wide variety of visual attributes including, amongst others, contrast^19^, orientation^20,21^ and motion^22,23^. In all of these cases, it has been shown that both the magnitude of the after-effect and its longevity depends on the duration of adaptation both psychophysically^24^ and neurophysiologically^25–27^. This is different from the deprivation-induced ocular dominance changes that we report here.

These findings may bear upon the clinical application of this form of plasticity in the adult for the restoration of balanced binocular function in amblyopia. Zhou *et al*.^4^ first showed that ocular dominance shifts from short term monocular deprivation also occur in adults with amblyopia and that they can be of larger magnitude and of a more sustained form. They suggested that short term occlusion of the amblyopic eye could be used to restore a more normal binocular balance. Such an approach would be the opposite of what is currently used in children, where the fixing eye rather than the amblyopic eye is patched. There are clinical trials currently underway to assess this novel approach (J. Zhou *et al*. and C. Lunghi, personal communications). The findings presented here are relevant in that it appears that the ocular dominance changes, at least in normal adults, are of an all-or-none form and, if this is also the case in amblyopes, this approach may be less suited to therapeutic intervention where long lasting effects are required. This can be contrasted with the dose-response (duration of patching) effect from monocular patching that is known to occur in children^28^. However, the present experiments have only involved single “pulses” of deprivation of varying duration, it is yet to be determined whether greater and more long-lasting summation changes in ocular dominance can be obtained from the interaction between double or indeed multiples “pulses” of monocular deprivation.

## Methods

### Participants

15 Adults (age = 24 ± 3 years) with normal or corrected-to-normal vision participated in this study. Two subjects were the listed first and third authors. All other subjects were naïve to the purpose of this study. We obtained an informed consent from the subjects. The study is in line with the Declaration of Helinski and was approved by the Institutional Review Boards at McGill University.

One cohort of eight subjects was patched for 60 and 180 minutes, another cohort of eight for 15 and 30 minutes. Four of the former cohort were also patched for 300 minutes. One of the authors participated in all experiments. All subjects completed two test sessions for every patching duration on different days.

### Apparatus

On a Mac computer, we employed Matlab 2012a and PsychToolBox 3.0.9 extensions to measure interocular sensory balance points of each subject in this study. We presented dichoptic stimuli using head mount goggles (eMagin Z800 pro, OLED) with a refresh rate of 60 Hz, resolution of 800 × 600 and mean luminance of 59 cd/m^2^. The goggles provided a linear input vs. luminance curve within the range of luminances used in the experiment.

### General Rationale

**Figure 5:**
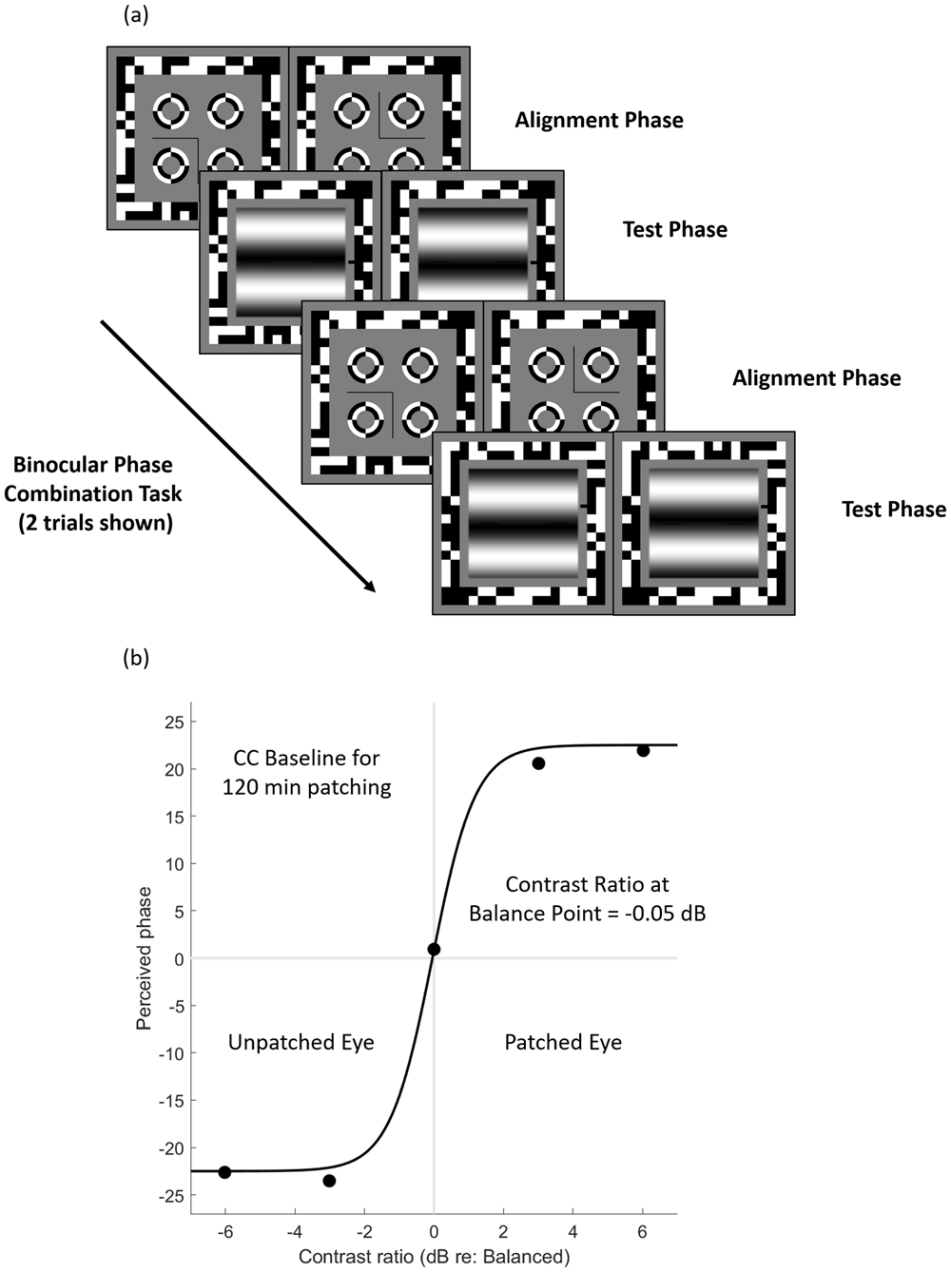
The temporal sequence of the binocular phase combination task and an illustration of fitting data to a binocular combination model. (**a**) Two trials of the binocular phase combination and two configurations are shown. There were 80 trials in the baseline test, and 30 trials in the post-patching test. The reference line was placed at the right side of the sinusoidal gratings. (**b**) Perceived phases from the binocular phase combination task during the baseline measurement of one subject were plotted as a function of contrast ratio. We fitted data from each measurement (baseline and post-patch) to a binocular combination model^30,31^.

We wanted to measure the contribution that each eye makes to the fused binocular percept. To do this each eye views a grating of each but opposite spatial phase (−22.5° for one eye and +22.5° for the other eye). If the contribution from each eye is equal then the binocularly fused percept will be of a grating of zero phase. If the contributions are not equal then the perceived phase can be reset to zero by offsetting the contrasts in each eye (see Fig. 5). The interocular contrast ratio that produces equal contribution (i.e. zero phase) is our measure of the ocular dominance.

### Stimuli

We used a binocular phase combination task as stimuli. A separate horizontal sine-wave grating (6.6° × 6.6° degrees of visual angle (deg), 0.3 cycles/deg) was presented to both dominant eye (DE) and non-dominant eye (NDE). The phases of the sinusoidal gratings were +22.5° in one eye and −22.5° in the other eye, both randomly assigned, relative to the center of the screen. Binocular presentation of these two gratings produced one fused grating percept. The phase difference between the gratings presented to both eyes ($\theta =|{\theta }_{DE}-{\theta }_{NDE}|$) was fixed at 45°. We measured the perceived phase of the fused grating at base contrast of 60%. In this study, we used the method of constant stimuli. The interocular contrast ratios between the eyes were 1/2, 1/$\sqrt{2}$, 1, $\sqrt{2}$, 2 in the baseline measurement test, and 1/$\sqrt{2}$, 1, $\sqrt{2}$ in the post-patch measurement test. There were 8 repetitions for every interocular ratio in baseline measurement, and 5 in post-patching measurement. The baseline test lasted for about 10 minutes and consisted of 80 trials whereas post-patching measurements lasted for about 3 minutes and consisted of 30 trials.

Two configurations were used to remove any starting positional bias (see Fig. 5a). The first configuration showed +22.5° to the dominant eye and −22.5° to the non-dominant eye, the second configuration −22.5° to the dominant eye and +22.5° to the non-dominant eye. We presented each configuration the same number of times in the task in a random order. Each configuration was repeated twice for every interocular ratio.

### Procedures

We patched subject’s dominant eye. To determine the dominant eye, we used the test described by Miles^29^. The subjects formed a peephole with their hands, stretched their peephole at arm’ length and located a target stimulus in the center of the peephole with both eyes open. They then alternately closed one eye and another and identified their dominant eye by determining when the object had most deviated from the center of the peephole.

**Figure 6:**
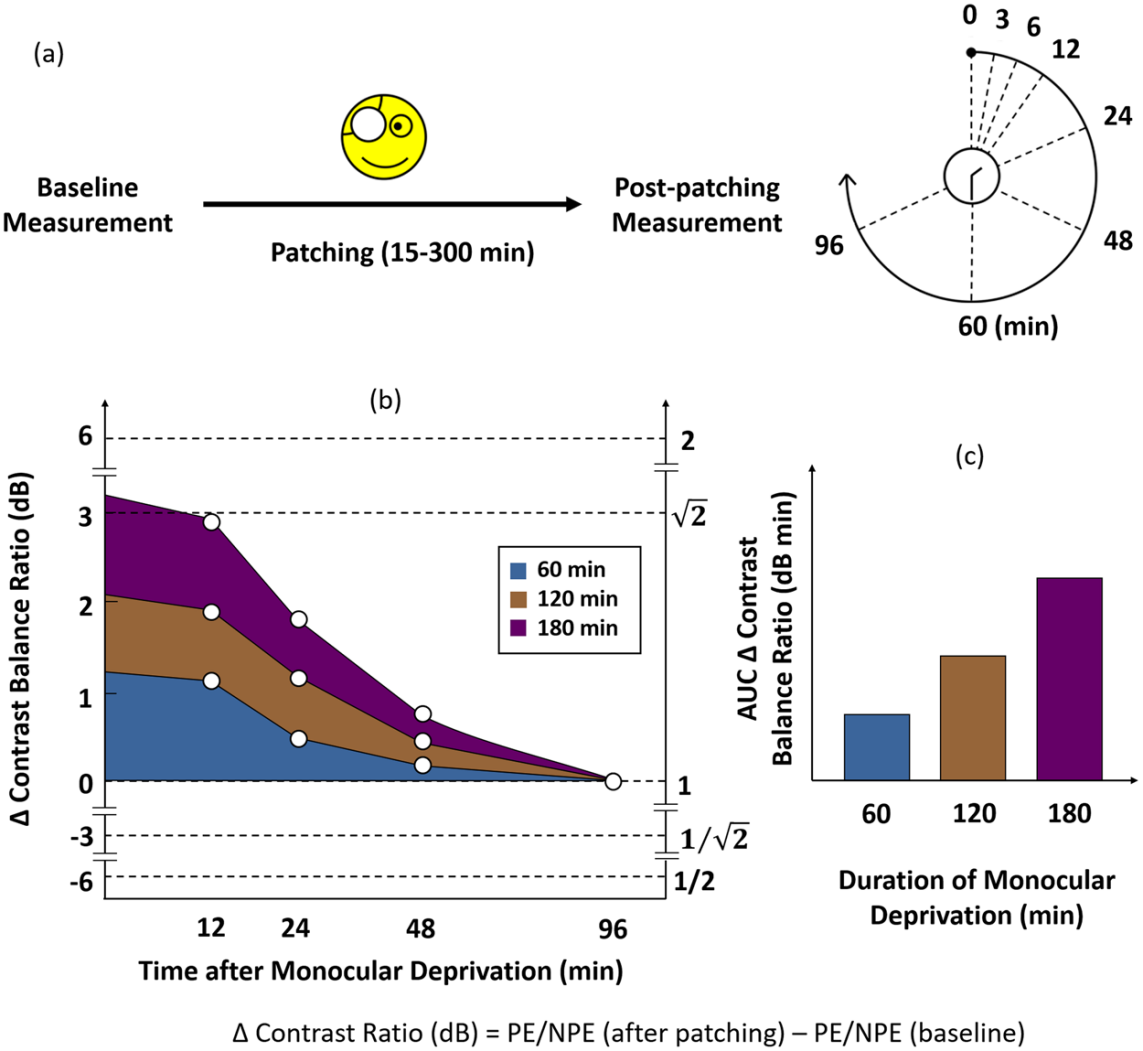
The temporal order for the experiments (**a**) and data analysis (**b**,**c**). Contrast balance ratio is defined by the contrast in the patched eye (PE) over that in the unpatched eye (NPE). (**a**) Time course of the experiment: Subjects performed a binocular phase combination task to measure their baseline eye balance. Their dominant eyes were patched for selected durations from 15 to 300 minutes. Finally, they performed the post-patching measurement with the same visual task at 0, 3, 6, 12, 24, 48, 60 and 96 minutes after patching. (**b**) Each point represents the difference in contrast balance ratios before and after monocular deprivation. 0 dB contrast balance ratio represents no difference between before and after monocular deprivation. Different colours represent different durations of monocular deprivation; the blue represents one hour, the brown two hours, and the purple three hours (**c**) After plotting the figure on the left we calculated the area under the curve (AUC) to quantity the overall effect of monocular deprivation; the higher the AUC, the greater the strengthening of the patched eye. The unit for AUC is dB minutes because the AUC is a product of two units (dB and minutes).

Before patching each subject completed the baseline test of binocular balance. Subjects performed two rounds of baseline measurement per session. They were then patched for certain durations with a translucent patch, which removes form information from the visual input and blocks some light transmission (20%)^11^. During patching, subjects either read a book or used a computer. After patch removal subjects performed the post-patching test at 0, 3, 6, 12, 24, 48, 60, and 96 minutes with a shorter version of the binocular phase combination task that they had performed for the baseline measurement (see Fig. 6a).

Throughout the combination task a pixelated binary noise frame was presented around the stimuli to encourage proper convergence (Fig. 5a). Before each trial, subjects performed an alignment procedure where the screens displayed a dichoptic cross enclosed by high-contrast circles. Subjects aligned the two halves of the cross and the circles using a keyboard. They then pressed the spacebar to begin the trial. The horizontal gratings appear to both eyes. Subjects were asked to move a reference line (thickness of one pixel) using the up and down keys of the keyboard to indicate the perceived center of the dark strip in the fused grating percept. They then pressed the spacebar to continue. The dichoptic cross then reappeared followed by the next trial (Fig. 5a).

### Analysis

We averaged the perceived phases from the two configurations. We then fitted the values to psychometric curves defined by a binocular phase combination model^30^ (Fig. 5b):

1 $${{\Phi }}_{A}=2ta{n}^{-1}\,[\frac{f(\alpha ,\,\beta ,\,\gamma )-{\delta }^{1+\gamma }}{f(\alpha ,\,\beta ,\,\gamma )+{\delta }^{1+\gamma }}\,tan(\frac{\theta }{2})],$$

where

2 $$f\,(\alpha ,\,\beta ,\,\gamma )=\frac{1+{\delta }^{\gamma }}{1+\alpha {\delta }^{\gamma }},$$

*θ* is the fixed phase difference between the gratings presented to both eyes (45°), Φ_*A*_ is the perceived phase of the two gratings, *δ* is the interocular contrast balance ratio (of the stimuli shown on the screen), *α* is a gain factor giving the contrast balance ratio between the two eyes when they contribute equally to binocular vision, and *γ* controls the slope of the transition between the left and right eye percepts. The two free parameters *α* and *γ* are estimated from fitting our data with the function. In our analysis, we bootstrapped the trial-by-trial responses of each to generate a bootstrapped population of *α* values for each measurement.

We converted *α* into log units using this equation:

3 $${\alpha }_{dB}=20\times lo{g}_{10}({\alpha }_{ratio}),$$

where

4 $${\alpha }_{ratio}=\frac{{\alpha }_{DE}}{{\alpha }_{NDE}}.$$

The estimated *α*_*ratio*_ represents contrast balance ratio when two eyes contribute equally to binocular vision on the linear scale. *α*_*dB*_ is the contrast balance ratio in log units. When *α*_*ratio*_ is 1, *α*_*dB*_ is 0, meaning that both eyes are equally balanced. If the patched eye is stronger than the unpatched eye, *α*_*ratio*_ > 1 and thus *α*_*dB*_ > 0. If the unpatched eye is stronger than the patched eye, 0 < *α*_*ratio*_ < 1 and thus *α*_*dB*_ > 0. The higher the value of *α*_*dB*_, the stronger the patched eye compared to the unpatched eye. We computed the difference between *α*_*dB*_ before and after monocular deprivation and plotted it as ∆ contrast balance ratio (see Fig. 6b, left frame for illustration) over time after monocular deprivation. Summary areal measures (units of dB minutes) were then derived (see Fig. 6c, right frame for illustration).
